# Supplementary material for: Can Drosophila melanogaster tell who’s who?
Source: PLoS One. 2018 Oct 24;13(10):e0205043. doi: 10.1371/journal.pone.0205043 (PMC6200205; doi:10.1371/journal.pone.0205043)
Supplement: S1 Methods — (PDF) [file pone.0205043.s001.pdf]

Can *Drosophila melanogaster* tell who's who?
Supplementary Methods

Connectome of the model fly-eye

Constructed from the published connectome [1]. We imposed a hierarchy (see text), but otherwise allowed links between 'lower' layers as long as the links were reported at least once (orange). Links between layers and connecting 'higher' levels were not used (blue). In brief, a 6-pixel filter is convolved through the image (representing photoreceptors R1-R6; whether or not the image is grayscale, the filter is fixed in all channels) and two additional colour-sensing filters are convolved (representing R7/R8; 1×1 pixel filter). The output of R1-R6 are then used as the feature map for lamina neurons L1-L5, which are locally connected 1×1 filters (i.e. different filters are learned at each spatial position). The outputs of these L1-L5s are locally convolved and are fed into the medulla intrinsic (Mi) neurons and/or the centrifugal (C) neurons, and/or the transmedullary (Tm) neurons. The C neurons feed into the Mi, Tm, T neurons. The Mi neurons feed into the Tm, and T neurons. The Tm neurons apply a filter and send their outputs to the T neurons. Sizes of the filters were determined from Takemura *et al.* [1], who traced connections through a single focal column (labelled Home) and up to two columns in any direction (in a hex grid they are labelled A-R). If a previous column had only connections to its respective column (i.e. Home→Home, A→A), it was modelled with a 1×1 locally-connected filter. If a previous column had more than 3 connections to its immediate surrounding columns (i.e. Home→A, C→D) then it was modelled with a 3×3 locally-connected filter. Finally, if it had more than 3 connections to more distant neighbours (i.e. Home→J, P→A), then it was modelled with a 5×5 locally-connected filter. Unfortunately, the connections between the medulla, lobula, and brain are not as documented as those between and within the lamina and medulla, but we implement a lobula neuron-like LC17 that concatenates Tm and T neurons with a 3×3 filter, while another neuron (LC4-like) concatenates Tm and T neurons with a 5×5 filter [2]. The output feature maps are then flattened and fed into two densely-connected layers with 256 neurons each before a soft-max layer.

CIFAR10 Data Processing

Images were standardized by subtracting the mean and dividing by the standard deviation of the training set for each colour channel. In all cases, to be comparable, the images were processed to trim a row and column from the top and left, and two rows and columns from the bottom and right (trimming to 29×29 pixels resulted in higher accuracy than resizing from 32×32) and were minimally augmented (random vertical flips and each image randomly offset by 3 pixels). For ResNet18 [3] and the Zeiler and Fergus [4] models, images were re-sized to 224×224.

CIFAR10 Results

The CIFAR-10 dataset consists of colour images (32×32 pixels) in 10 classes (airplane, automobile, bird, cat, deer, dog, frog, horse, ship, and truck) [5]. The current state of the art models can achieve 97.44% accuracy (with clever data augmentations [6]), while human performance has been estimated at around 94% accuracy [7]. Our re-implementation of ResNet18 [3] achieves 0.91 (F_1 score). The Zeiler and Fergus model [4], that has been shown to rival the representational performance of the human inferior temporal cortex [8] (Illustrated in Figure 2A), achieves a lower F_1 score of 0.85, revealing the gap between the ability to represent mid-level complexity and highest order syntactic information. Our simplified implementation of the fly visual system achieves 0.58. The CIFAR10 results are summarized in Supplementary Table 2.

References

1. Takemura Sy, Nern A, Chklovskii DB, Scheffer LK, Rubin GM, Meinertzhagen IA. The comprehensive connectome of a neural substrate for ‘ON’motion detection in Drosophila. Elife. 2017;6.

2. Wu M, Nern A, Williamson WR, Morimoto MM, Reiser MB, Card GM, et al. Visual projection neurons in the Drosophila lobula link feature detection to distinct behavioral programs. Elife. 2016;5.

3. He K, Zhang X, Ren S, Sun J. Deep residual learning for image recognition. In: Proceedings of the IEEE conference on computer vision and pattern recognition; 2016. p. 770–778.

4. Zeiler MD, Fergus R. Visualizing and understanding convolutional networks. In: European conference on computer vision. Springer; 2014. p. 818–833.

5. Krizhevsky A. Learning multiple layers of features from tiny images. 2009;.

6. DeVries T, Taylor GW. Improved regularization of convolutional neural networks with cutout. arXiv preprint arXiv:170804552. 2017;.

7. Karpathy A. Lessons learned from manually classifying CIFAR-10; 2011.

8. Cadieu CF, Hong H, Yamins DL, Pinto N, Ardila D, Solomon EA, et al. Deep neural networks rival the representation of primate IT cortex for core visual object recognition. PLoS computational biology. 2014;10(12):e1003963.
