# Supplementary figures and images for: Can Drosophila melanogaster tell who’s who?
Source: PLoS One. 2018 Oct 24;13(10):e0205043. doi: 10.1371/journal.pone.0205043 (PMC6200205; doi:10.1371/journal.pone.0205043)

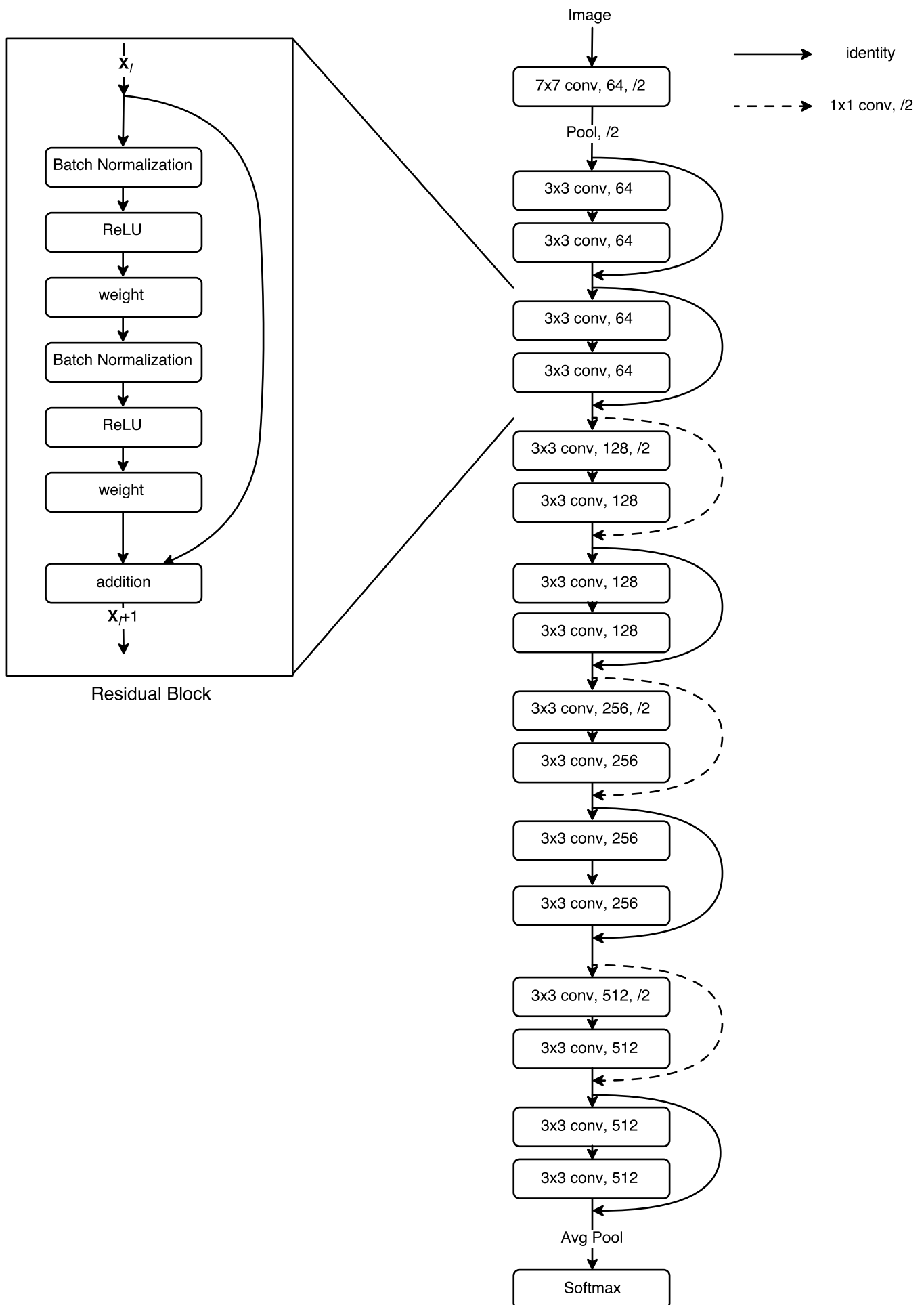

Supplement: S1 Fig — Residual Networks with 18 ‘layers’ [19] were constructed as depicted, using the improved block scheme proposed by [19] (top left inset). (PDF) [file pone.0205043.s002.pdf]

Data examples

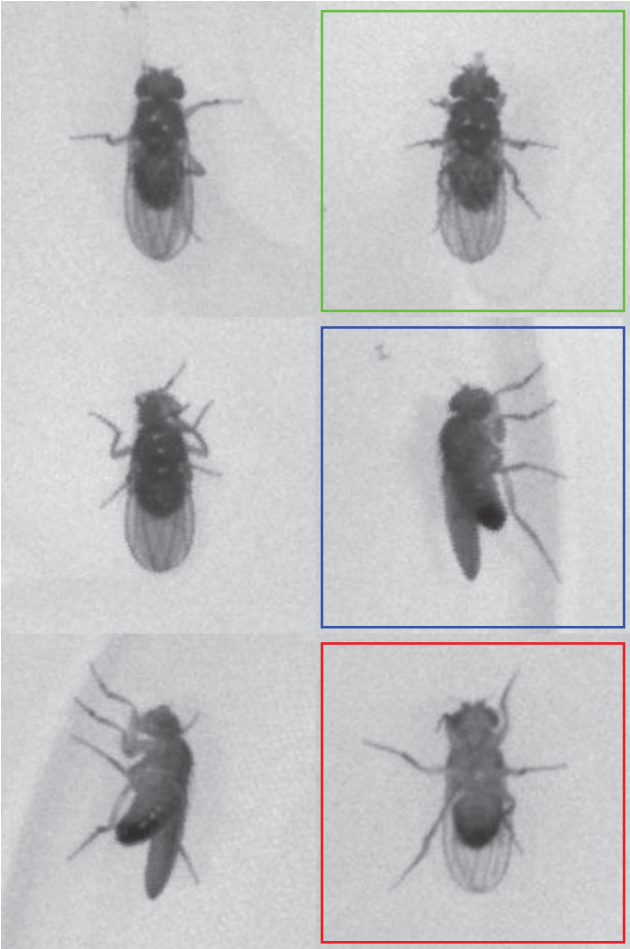

Augmented data examples

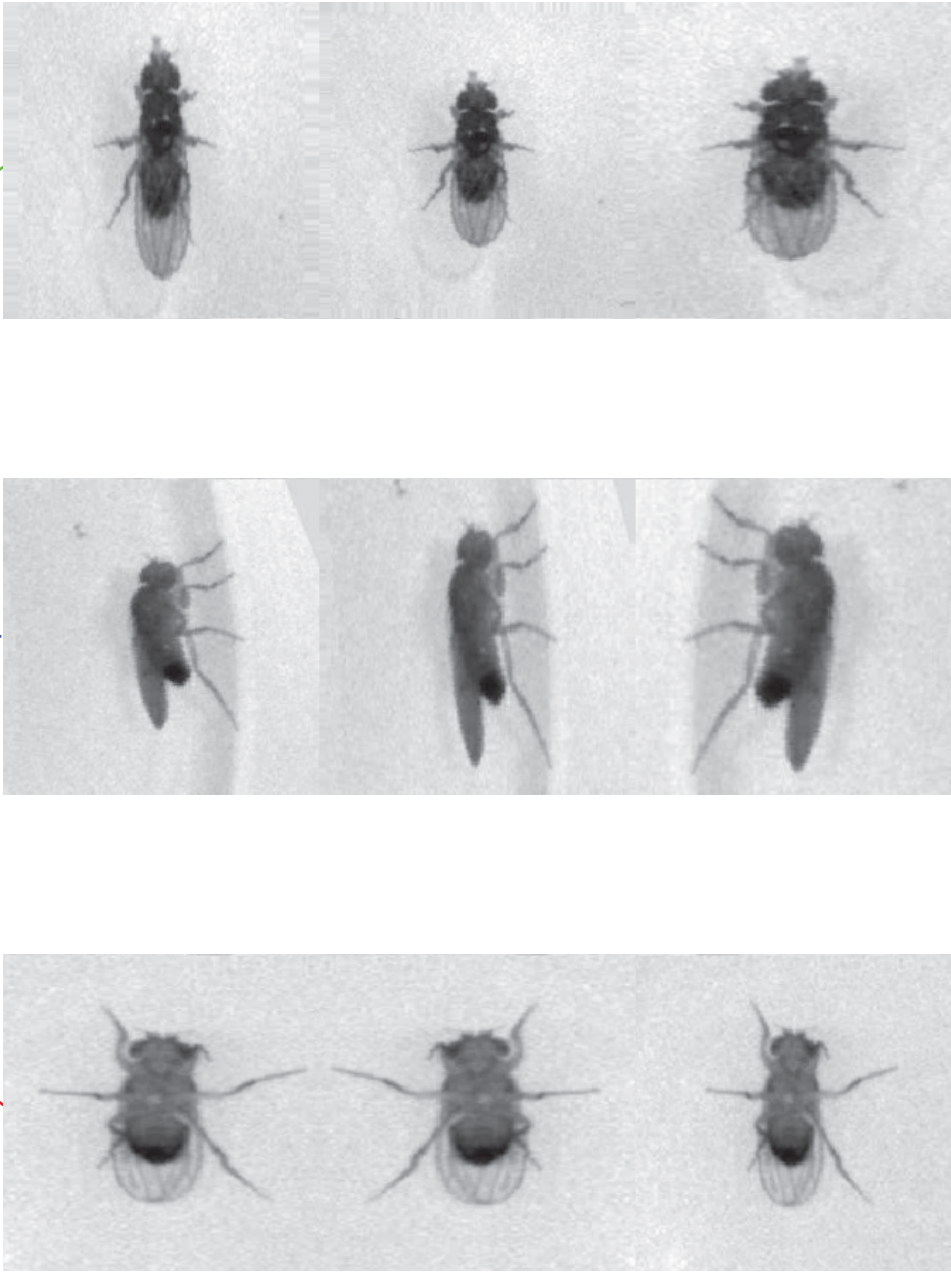

Supplement: S2 Fig — On the left are examples of the various viewpoints of the fly. The images indicated were run through the random non-proportion-preserving zoom to generate three examples of each. (PDF) [file pone.0205043.s003.pdf]

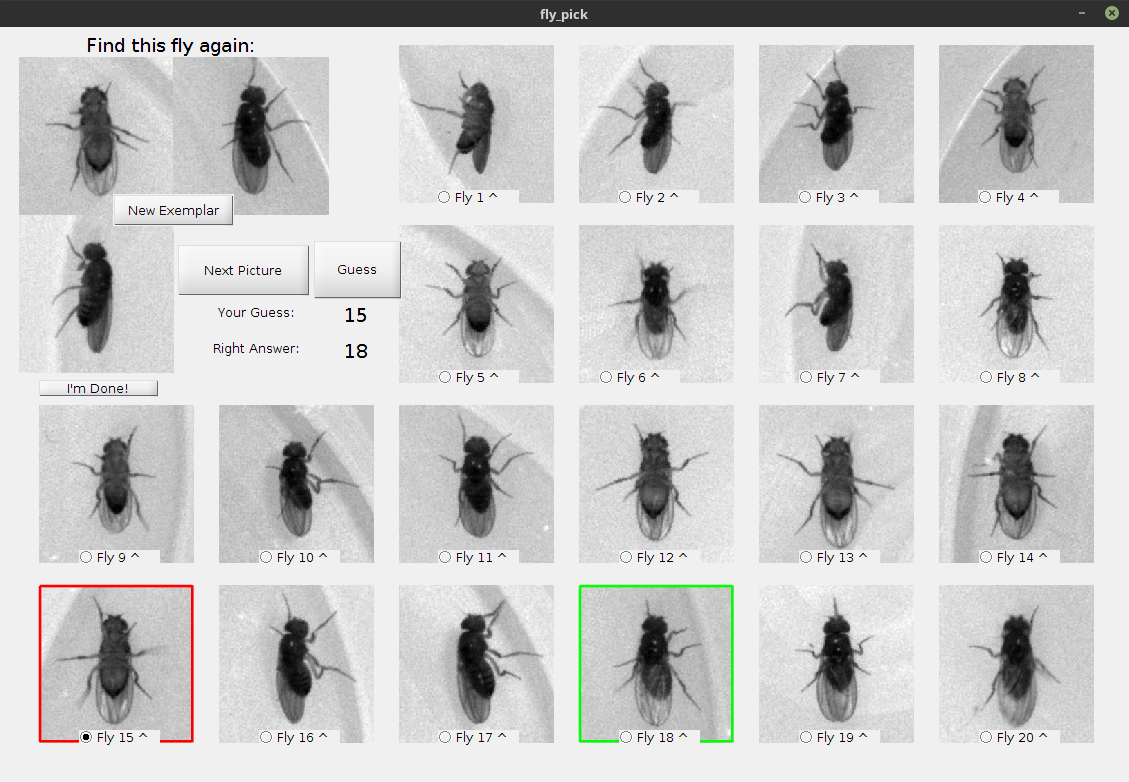

Supplement: S3 Fig — Participants were asked to re-identify the exemplar fly (top left), which exhibited three viewpoints: ventral, dorsal, and angled. They could request new images of said fly as many times as needed. The exemplar images were manually sorted into viewpoint categories and then randomly selected from Days 1 and 2. The other fly images (queries) were selected from Day 3. (PNG) [file pone.0205043.s004.png]

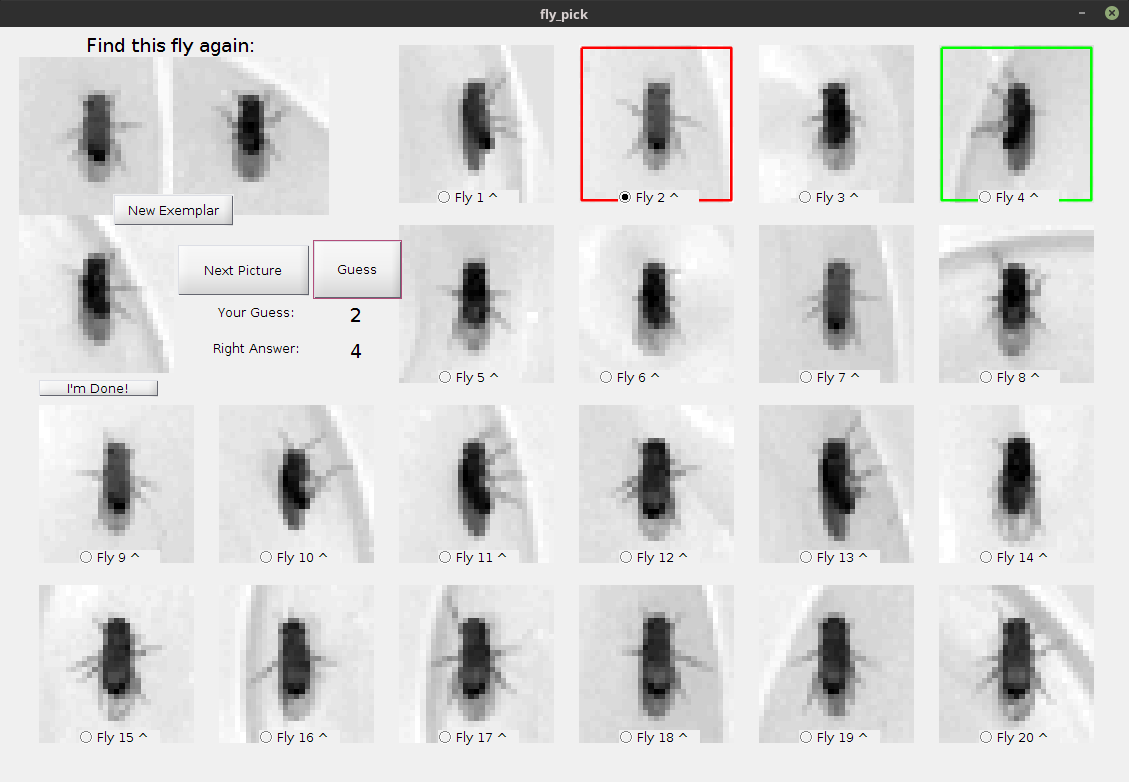

Supplement: S4 Fig — Similar to the high-resolution experiment described above (S3 Fig), except all pictures were down sampled to 29×29. (PNG) [file pone.0205043.s005.png]
