# Supplementary material for: Can Drosophila melanogaster tell who’s who?
Source: PLoS One. 2018 Oct 24;13(10):e0205043. doi: 10.1371/journal.pone.0205043 (PMC6200205; doi:10.1371/journal.pone.0205043)
Supplement: S1 Table — The table indicates the filter size for the locally-connected layers. No self connections were allowed (grey), or connections with ‘higher’ layers (blue). See S1 Methods for additional details. (PDF) [file pone.0205043.s006.pdf]

Table S1 Model fly-eye connectome.

	R1-6	R7	R8	L1	L2	L3	L4	L5	C2	C3	Mi1	Mi4	Mi9	Mi15	Tm20	Tm1	Tm2	Tm3	Tm4	Tm6	Tm9	TmY5a	T2a	T2	T3
R1-6	0	0	0	1	1	1	1	1	0	0	0	0	0	0	0	0	0	0	0	0	0	0	0	0	0
R7	0	0	1	0	0	1	0	0	0	0	0	0	1	0	1	0	0	0	0	0	0	0	0	0	0
R8	0	1	0	1	0	1	0	1	0	0	1	1	1	3	1	0	0	0	0	0	0	0	0	0	0
L1	0	0	0	0	0	0	0	1	1	1	1	0	0	0	0	0	0	5	0	0	0	0	0	0	0
L2	0	0	0	0	0	0	0	1	0	1	0	1	1	0	1	1	1	0	5	0	0	0	1	0	3
L3	0	0	0	0	0	0	0	0	0	1	1	1	3	0	0	0	0	3	0	0	1	0	0	0	0
L4	0	0	0	0	0	0	0	0	0	0	0	0	3	0	0	0	3	0	3	0	3	0	0	0	3
L5	0	0	0	1	1	0	0	3	5	3	3	3	0	3	0	1	0	5	0	5	0	3	1	0	3
C2	0	0	0	1	1	1	3	1	0	1	1	1	0	0	0	1	0	3	0	0	1	0	1	0	3
C3	0	0	0	0	1	0	0	1	0	0	1	1	0	0	1	1	1	0	5	0	1	0	1	0	3
Mi1	0	0	0	1	0	0	0	0	5	1	1	3	1	3	1	1	0	5	0	3	0	0	0	3	3
Mi4	0	0	0	0	1	0	0	0	0	0	1	3	1	0	3	1	1	3	3	0	3	3	1	0	0
Mi9	0	0	0	0	0	0	0	0	0	0	0	1	0	1	0	1	1	3	3	5	0	3	0	0	3
Mi15	0	0	0	0	0	0	0	0	0	3	0	3	0	5	0	0	0	0	0	3	0	0	0	0	0
Tm20	0	0	0	0	0	0	0	0	0	0	0	0	1	0	3	0	0	0	0	0	0	0	0	0	0
Tm1	0	0	0	0	1	0	0	1	0	3	0	1	1	0	1	0	1	0	3	5	1	0	1	3	3
Tm2	0	0	0	0	1	0	0	1	0	3	1	1	3	0	0	1	0	0	3	5	0	0	1	0	3
Tm3	0	0	0	3	0	0	0	0	0	3	3	3	3	0	0	0	0	5	0	0	0	5	0	3	3
Tm4	0	0	0	0	0	0	0	0	0	3	0	3	0	0	0	0	0	0	3	3	0	3	0	3	0
Tm6	0	0	0	0	0	0	0	0	0	0	0	0	0	0	0	0	0	0	0	3	0	5	0	0	3
Tm9	0	0	0	0	0	0	0	0	0	0	0	0	1	0	1	0	0	0	0	0	0	0	0	0	0
TmY5a	0	0	0	0	0	0	0	0	0	0	0	0	0	0	0	0	0	0	0	5	0	0	0	3	3
T2a	0	0	0	0	1	0	0	1	0	0	0	0	0	0	1	0	1	0	0	0	0	0	0	0	0
T2	0	0	0	0	0	0	0	0	0	0	3	0	0	0	3	0	0	0	0	0	0	3	0	0	0
T3	0	0	0	0	0	0	0	0	0	0	3	0	0	0	0	0	0	0	0	3	0	3	0	0	3
