# Supplementary material for: Can Drosophila melanogaster tell who’s who?
Source: PLoS One. 2018 Oct 24;13(10):e0205043. doi: 10.1371/journal.pone.0205043 (PMC6200205; doi:10.1371/journal.pone.0205043)
Supplement: S2 Table — See S1 Methods for additional information about data processing and results. (PDF) [file pone.0205043.s007.pdf]

S2 Table Results of a simple vision task (CIFAR10).

**Model Name**	**'Neurons' (#)**	**Parameters (#)**	**Accuracy (F_1 Score)[1]**
Human Performance	Billions		0.94[2]
ResNet18	˜2 million	˜11 million	0.9148[3]
Zeiler and Fergus	˜1.6 million	˜72 million	0.8488[3]
**Our fly-eye**	25,742	1,350,316	**0.5364**
Random Chance			0.10

[1] The F_1 score combines precision (probability of assigning the right ID to the right class) and recall (probability that the ID assigned is to the right class).
[2] Human performance is given in overall accuracy (%).
[3] Images were re-sized from 29×29 to 224× 224. Their results presented here are not for state-of-the-art benchmarking, but for comparison.
