# Supplementary material for: Can Drosophila melanogaster tell who’s who?
Source: PLoS One. 2018 Oct 24;13(10):e0205043. doi: 10.1371/journal.pone.0205043 (PMC6200205; doi:10.1371/journal.pone.0205043)
Supplement: S4 Table — Note that fly 10 has a recall accuracy of 37%, and is almost equally confused for flies 2 and 7. Flies are ordered by sex (Purple = male, Yellow = Female), then by ascending size. Predictions are colour coded and weighted by percentage (correct predictions are indicated in orange, incorrect predictions are coloured cyan). (PDF) [file pone.0205043.s009.pdf]

S4 Table Confusion Matrix for ResNet18 Biological Replicate 2.

ID	1	2	7	3	10	9	6	8	4	5	13	14	20	18	16	17	15	11	19	12
1	93	0	1	0	0	3	1	1	1	0	0	0	0	0	0	0	0	0	0	0
2	0	98	1	0	0	0	0	0	0	0	0	0	0	0	0	0	0	0	0	0
7	0	8	89	0	1	2	0	0	0	0	0	0	0	0	0	0	0	0	0	0
3	0	0	0	99	0	0	0	0	0	0	0	0	0	0	0	0	0	0	0	0
10	0	29	27	1	37	1	0	1	2	1	0	0	0	0	0	0	0	0	0	0
9	0	0	0	0	0	99	0	0	0	0	0	0	0	0	0	0	0	0	0	0
6	0	1	0	0	0	0	98	0	1	0	0	0	0	0	0	0	0	0	0	0
8	0	0	0	0	0	0	0	99	0	0	0	0	0	0	0	0	0	0	0	0
4	0	0	0	0	0	0	0	0	99	0	0	0	0	0	0	0	0	0	0	0
5	1	1	2	0	0	3	2	3	4	82	0	0	0	0	0	0	0	0	0	0
13	0	0	0	0	0	0	0	0	0	0	95	1	1	2	0	2	0	0	0	0
14	0	0	0	0	0	0	0	0	0	0	1	98	0	1	0	0	0	0	0	0
20	0	0	0	0	0	0	0	0	0	0	0	0	97	0	0	1	0	0	0	0
18	0	0	0	0	0	0	0	0	0	0	0	0	0	97	0	1	0	0	0	2
16	0	0	0	0	0	0	0	0	0	0	0	0	0	2	70	0	0	4	0	24
17	0	0	0	0	0	0	0	0	0	0	0	0	0	3	0	96	0	0	0	0
15	0	0	0	0	0	0	0	0	0	0	0	0	0	5	0	0	93	0	0	1
11	0	0	0	0	0	0	0	0	0	0	0	0	0	0	0	0	0	99	0	1
19	0	0	0	0	0	0	0	0	0	0	1	6	0	1	1	7	0	1	82	2
12	0	0	0	0	0	0	0	0	0	0	0	0	0	1	0	0	0	0	0	98
