# Supplementary material for: Can Drosophila melanogaster tell who’s who?
Source: PLoS One. 2018 Oct 24;13(10):e0205043. doi: 10.1371/journal.pone.0205043 (PMC6200205; doi:10.1371/journal.pone.0205043)
Supplement: S5 Table — Flies are ordered by sex (Purple = male, Yellow = Female), then by ascending size. Predictions are colour coded and weighted by percentage (correct predictions are indicated in orange, incorrect predictions are coloured cyan). (PDF) [file pone.0205043.s010.pdf]

S5 Table   Confusion Matrix for ResNet18 Biological Replicate 3.

ID	1	2	5	7	3	10	9	4	6	8	18	19	14	15	11	16	20	17	13	12
1	71	1	2	0	1	22	0	1	1	0	0	0	0	0	0	0	0	0	0	0
2	0	99	0	0	0	0	0	0	0	0	0	0	0	0	0	0	0	0	0	0
5	0	6	92	0	0	0	0	1	1	0	0	0	0	0	0	0	0	0	0	0
7	0	0	0	89	7	0	0	0	0	4	0	0	0	0	0	0	0	0	0	0
3	0	0	0	1	96	1	0	0	0	1	0	0	0	0	0	0	0	0	0	0
10	0	0	1	0	0	97	0	1	0	0	0	0	0	0	0	0	0	0	0	0
9	2	3	0	0	0	0	94	1	1	0	0	0	0	0	0	0	0	0	0	0
4	0	0	2	0	0	0	0	97	0	0	0	0	0	0	0	0	0	0	0	0
6	0	1	6	0	1	0	0	1	90	0	0	0	0	0	0	0	0	0	0	0
8	0	0	0	0	4	1	0	1	0	94	0	0	0	0	0	0	0	0	0	0
18	0	0	0	0	0	0	0	0	0	0	99	0	0	0	0	0	0	0	0	0
19	0	0	0	0	0	0	0	0	0	0	1	95	0	0	0	0	0	3	0	0
14	0	0	0	0	0	0	0	0	0	0	0	0	99	0	0	0	1	0	0	0
15	0	0	0	0	0	0	0	0	0	0	0	0	0	99	0	0	0	0	0	0
11	0	0	0	0	0	0	0	0	0	0	0	0	0	0	99	0	0	0	0	0
16	0	0	0	0	0	0	0	0	0	0	0	0	0	0	0	100	0	0	0	0
20	0	0	0	0	0	0	0	0	0	0	0	0	2	0	0	0	97	0	0	0
17	0	0	0	0	0	0	0	0	0	0	0	0	0	0	0	0	0	100	0	0
13	0	0	0	0	0	0	0	0	0	0	0	0	0	2	0	1	0	1	96	0
12	0	0	0	0	0	0	0	0	0	0	0	0	0	0	0	0	2	0	0	97
