# Supplementary material for: Can Drosophila melanogaster tell who’s who?
Source: PLoS One. 2018 Oct 24;13(10):e0205043. doi: 10.1371/journal.pone.0205043 (PMC6200205; doi:10.1371/journal.pone.0205043)
Supplement: S6 Table — Flies are ordered by sex (Purple = male, Yellow = Female), then by ascending size. Predictions are colour coded and weighted by percentage (correct predictions are indicated in orange, incorrect predictions are coloured cyan). (PDF) [file pone.0205043.s011.pdf]

S6 Table Confusion Matrix for Fly-Eye Model Biological Replicate 1.

ID	1	10	3	8	2	6	4	9	7	5	11	18	15	14	19	17	20	13	16	12
1	86	1	5	1	1	0	4	0	2	0	0	0	0	0	0	0	0	0	0	0
10	0	93	0	0	0	0	5	0	1	0	0	0	0	0	0	0	0	0	0	0
3	4	0	74	4	6	1	3	2	7	0	0	0	0	0	0	0	0	0	0	0
8	2	0	16	69	5	1	1	2	4	0	0	0	0	0	0	0	0	0	0	0
2	0	0	6	2	73	7	0	7	3	0	0	0	0	0	0	0	0	0	0	0
6	0	0	12	2	2	71	0	12	1	1	0	0	0	0	0	0	0	0	0	0
4	4	2	4	1	0	0	84	0	4	0	0	0	0	0	0	0	0	0	0	0
9	0	0	2	2	7	3	0	81	2	2	0	0	0	0	0	0	0	0	0	0
7	1	1	6	1	4	0	2	0	84	0	0	0	0	0	0	0	0	0	0	0
5	0	0	0	0	2	4	0	2	0	91	0	0	0	0	0	0	0	0	0	0
11	0	0	0	0	0	0	0	0	0	0	89	0	0	4	1	2	0	4	0	0
18	0	0	0	0	0	0	0	0	0	0	0	90	4	0	0	0	3	0	2	0
15	0	0	0	0	0	0	0	0	0	0	1	1	93	1	0	2	0	0	1	0
14	0	0	0	0	0	0	0	0	0	0	1	1	2	90	0	0	0	1	4	0
19	0	0	0	0	0	0	0	0	0	0	0	0	0	0	97	0	0	0	0	2
17	0	0	0	0	0	1	0	0	0	1	3	0	1	0	0	93	0	0	0	1
20	0	0	0	0	0	0	0	0	0	0	0	3	2	0	0	0	92	0	4	0
13	0	0	0	0	0	0	0	0	0	0	3	0	0	4	0	0	0	91	1	0
16	0	0	0	0	0	0	0	0	0	0	0	2	2	5	0	0	1	0	89	0
12	0	0	0	0	0	0	0	0	0	0	0	0	1	1	1	0	0	0	0	96
