# Supplementary material for: Can Drosophila melanogaster tell who’s who?
Source: PLoS One. 2018 Oct 24;13(10):e0205043. doi: 10.1371/journal.pone.0205043 (PMC6200205; doi:10.1371/journal.pone.0205043)
Supplement: S7 Table — Flies are ordered by sex (Purple = male, Yellow = Female), then by ascending size. Predictions are colour coded and weighted by percentage (correct predictions are indicated in orange, incorrect predictions are coloured cyan). (PDF) [file pone.0205043.s012.pdf]

S7 Table Confusion Matrix for Fly-Eye Model Biological Replicate 2.

ID	1	2	7	3	10	9	6	8	4	5	13	14	20	18	16	17	15	11	19	12
1	62	1	4	0	2	9	3	6	2	12	0	0	0	0	0	0	0	0	0	0
2	2	63	10	5	12	2	1	0	5	2	0	0	0	0	0	0	0	0	0	0
7	3	9	60	1	11	6	0	1	1	8	0	0	0	0	0	0	0	0	0	0
3	0	15	2	72	4	0	2	0	4	0	0	0	0	0	0	0	0	0	0	0
10	5	11	27	1	38	5	2	1	7	3	0	0	0	0	0	0	0	0	0	0
9	1	4	4	0	1	82	1	1	3	2	0	0	0	0	0	0	0	0	0	0
6	3	5	4	3	5	1	65	1	9	3	0	0	0	0	0	0	0	0	0	0
8	4	0	1	0	0	7	0	81	0	5	0	0	0	0	0	0	0	0	0	0
4	1	2	1	1	4	6	1	0	82	2	0	0	0	0	0	0	0	0	0	0
5	5	5	9	1	4	21	3	3	9	41	0	0	0	0	0	0	0	0	0	0
13	0	0	0	0	0	0	0	0	0	0	54	22	8	5	0	10	0	0	0	0
14	0	0	0	0	0	0	0	0	0	0	2	88	3	1	0	5	1	0	0	0
20	0	0	0	0	0	0	0	0	0	0	2	8	57	3	0	30	0	0	0	1
18	0	0	0	0	0	0	0	0	0	0	0	1	1	43	0	51	0	0	0	4
16	0	0	0	0	0	0	0	0	0	0	0	0	0	6	32	1	13	16	3	29
17	0	0	0	0	0	0	0	0	0	0	0	2	4	6	0	86	0	0	0	1
15	0	0	0	0	0	0	0	0	0	0	3	2	1	6	1	1	84	0	0	2
11	0	0	0	0	0	0	0	0	0	0	0	0	0	0	4	0	1	90	1	4
19	0	0	0	0	0	0	0	0	0	0	4	1	1	17	1	35	2	1	36	2
12	0	0	0	0	0	0	0	0	0	0	0	0	1	27	3	6	1	1	1	60
