# Supplementary material for: Can Drosophila melanogaster tell who’s who?
Source: PLoS One. 2018 Oct 24;13(10):e0205043. doi: 10.1371/journal.pone.0205043 (PMC6200205; doi:10.1371/journal.pone.0205043)
Supplement: S8 Table — Flies are ordered by sex (Purple = male, Yellow = Female), then by ascending size. Predictions are colour coded and weighted by percentage (correct predictions are indicated in orange, incorrect predictions are coloured cyan). (PDF) [file pone.0205043.s013.pdf]

S8 Table Confusion Matrix for Fly-Eye Model Biological Replicate 3.

ID	1	2	5	7	3	10	9	4	6	8	18	19	14	15	11	16	20	17	13	12
1	27	1	2	0	2	55	2	6	5	1	0	0	0	0	0	0	0	0	0	0
2	0	84	11	0	0	1	1	1	3	0	0	0	0	0	0	0	0	0	0	0
5	1	7	78	0	0	2	1	5	6	0	0	0	0	0	0	0	0	0	0	0
7	0	0	0	59	26	2	0	1	0	11	0	0	0	0	0	0	0	0	0	0
3	1	0	0	12	65	7	0	1	0	12	0	0	0	0	0	0	0	0	0	0
10	3	0	1	3	8	73	0	3	3	3	0	0	0	0	0	0	0	0	0	0
9	4	11	4	0	0	0	71	3	8	0	0	0	0	0	0	0	0	0	0	0
4	0	0	5	0	1	4	0	88	1	0	0	0	0	0	0	0	0	0	0	0
6	3	3	11	0	1	10	3	6	63	0	0	0	0	0	0	0	0	0	0	0
8	1	0	0	8	22	12	0	2	0	55	0	0	0	0	0	0	0	0	0	0
18	0	0	0	0	0	0	0	0	0	0	89	6	2	0	1	0	3	0	0	0
19	0	0	0	0	0	0	0	0	0	0	5	87	0	1	1	0	0	1	4	1
14	0	0	0	0	0	0	0	0	0	0	0	1	96	0	0	0	1	0	0	0
15	0	0	0	0	0	0	0	0	0	0	0	1	0	94	1	0	0	1	4	0
11	0	0	0	0	0	0	0	0	0	0	2	0	2	0	92	0	1	0	0	3
16	0	0	0	0	0	0	0	0	0	0	0	0	0	0	0	98	0	0	0	1
20	0	0	0	0	0	0	0	0	0	0	2	0	2	0	0	2	92	0	0	2
17	0	0	0	0	0	0	0	0	0	0	0	2	0	1	0	0	0	77	20	0
13	0	0	0	0	0	0	0	0	0	0	0	0	0	15	0	2	0	8	70	4
12	0	0	0	0	0	0	0	0	0	0	5	0	0	0	2	1	9	0	1	80
